# Phytoplasma-conserved phyllogen proteins induce phyllody across the Plantae by degrading floral MADS domain proteins

**DOI:** 10.1093/jxb/erx158

**Published:** 2017-05-15

**Authors:** Yugo Kitazawa, Nozomu Iwabuchi, Misako Himeno, Momoka Sasano, Hiroaki Koinuma, Takamichi Nijo, Tatsuya Tomomitsu, Tetsuya Yoshida, Yukari Okano, Nobuyuki Yoshikawa, Kensaku Maejima, Kenro Oshima, Shigetou Namba

**Affiliations:** 1 Department of Agricultural and Environmental Biology, Graduate School of Agricultural and Life Sciences, The University of Tokyo, Yayoi, Bunkyo-ku, Tokyo, Japan; 2 Faculty of Agriculture, Iwate University, 3-18-8 Ueda, Morioka-shi, Iwate, Japan; 3 Faculty of Bioscience, Hosei University, 3-7-2 Kajino-cho, Koganei-shi, Tokyo, Japan

**Keywords:** ABCE model, floral development, MADS domain transcription factor, phyllody, phyllogen, phytoplasma

## Abstract

ABCE-class MADS domain transcription factors (MTFs) are key regulators of floral organ development in angiosperms. Aberrant expression of these genes can result in abnormal floral traits such as phyllody. Phyllogen is a virulence factor conserved in phytoplasmas, plant pathogenic bacteria of the class Mollicutes. It triggers phyllody in *Arabidopsis thaliana* by inducing degradation of A- and E-class MTFs. However, it is still unknown whether phyllogen can induce phyllody in plants other than *A. thaliana*, although phytoplasma-associated phyllody symptoms are observed in a broad range of angiosperms. In this study, phyllogen was shown to cause phyllody phenotypes in several eudicot species belonging to three different families. Moreover, phyllogen can interact with MTFs of not only angiosperm species including eudicots and monocots but also gymnosperms and a fern, and induce their degradation. These results suggest that phyllogen induces phyllody in angiosperms and inhibits MTF function in diverse plant species.

## Teaser

Phyllogen, a bacterial virulence factor, induced phyllody in various eudicot species, and had broad-spectrum degradation activity on MADS domain transcription factors of plants, suggesting phyllogen universally functions in plants.

## Introduction

Flowers exhibit a wide variety of shapes and colors, and sometimes have abnormal phenotypes (Meyer, 1966), including phyllody (replacement of floral organs by leaf-like structures) and double flowers (flowers with extra petals or petal-like structures). Plants with such abnormal flower phenotypes have often been used as commercial cultivars (Kanno, 2016) and genetic resources to study molecular mechanisms of floral development (Meyerowitz *et al.*, 1989; Wellmer *et al.*, 2014). In model eudicots such as *Arabidopsis thaliana*, the identity of floral organs is regulated by floral homeotic genes divided into ABCE-classes based on function (the ABCE-model): A- and E-class genes specify sepal identity; A-, B-, and E-class genes petal identity; B-, C-, and E-class genes stamen identity; and C- and E-class genes carpel identity (Krizek and Fletcher, 2005; Soltis *et al.*, 2007; Rijpkema *et al.*, 2010). ABCE-class genes mostly encode type II MADS domain transcription factors (MTFs), which are thought to form quaternary complexes in each floral organ (the floral quartet model; Honma and Goto, 2001; Theißen *et al.*, 2016). Homologs of ABCE-class genes are often found in angiosperms (Zahn *et al.*, 2005*a*, *b*; Shan *et al.*, 2007; Dreni and Kater, 2014), and the functions of B-, C-, and E-class MTFs are thought to be conserved (Cui *et al.*, 2010; Heijmans *et al.*, 2012*b*; Wang *et al.*, 2015). Thus, the ABCE model is considered to be applicable in diverse angiosperms, especially core eudicots (Soltis *et al.*, 2007), although differences between plant species have been observed (Rijpkema *et al.*, 2010; Yoshida and Nagato, 2011; Teixeira da Silva *et al.*, 2014).

Aberrant expression of the MTFs can alter floral organ identity and morphology, and result in the production of abnormal flowers in many plant species. For example, gene knockdown or knockout of C-class MTFs leads to the development of petal-like stamens in model plants, such as *A. thaliana*, petunia (*Petunia*×*hybrida*), and *Antirrhinum majus* (Yanofsky, 1990; Bradley *et al.*, 1993; Heijmans *et al.*, 2012*a*). Other reports have also shown that down-regulation of E-class MTFs leads to the replacement of all or partial flower organs by leaf-like structures in *A. thaliana,* petunia, and rice (Angenent *et al.*, 1994; Pelaz *et al.*, 2000; Vandenbussche *et al.*, 2003; Ditta *et al.*, 2004; Cui *et al.*, 2010). Additionally, it has been reported that MTF genes are aberrantly expressed in several horticultural varieties with unique flower characteristics (Sun *et al.*, 2014; Yan *et al.*, 2016).

Phytoplasmas (‘*Candidatus* Phytoplasma’ spp.) are phloem-limited plant pathogenic bacteria that infect hundreds of plant species. They are capable of inducing various symptoms, including abnormal development of flowers such as phyllody, virescence (green coloration of floral organs), and a loss of floral meristem determinacy (production of stem-like pistils) (Lee *et al.*, 2000; Arashida *et al.*, 2008; Chaturvedi *et al.*, 2010; Maejima *et al.*, 2014*b*). Such flower malformations associated with phytoplasmas are widely observed in both eudicot and monocot species (Chaturvedi *et al.*, 2010), and sometimes considered very attractive and valuable (Wang and Maramorosch, 1988; Strauss, 2009). Recently, it was shown that a family of highly conserved phytoplasma virulence factors, designated as a phyllody-inducing gene (phyllogen) family, induces phyllody and other floral malformation in *A. thaliana* (MacLean *et al.*, 2011; Maejima *et al.*, 2014*a*; Yang *et al.*, 2015). PHYL1_OY_, a phyllogen from the ‘*Ca.* P. asteris’ onion yellows strain (OY), interacts directly with APETALA1 (AP1) and SEPALLATA1 (SEP1)–SEP4 (A- and E-class MTFs, respectively) of *A. thaliana* (Maejima *et al.*, 2014*a*, 2015), and induces their degradation in a proteasome-dependent manner (Maejima *et al.*, 2014*a*). Another phyllogen, SAP54, was also reported to bind to and induce degradation of A- and E-class MTFs, but it did not bind to APETALA3 (AP3) and PISTILLATA (PI; B-class MTFs), or to AGAMOUS (AG; C-class MTF; MacLean *et al.* 2014). Therefore, phyllogen-mediated degradation of the A- and E-class MTFs is regarded as the molecular mechanism responsible for phyllody symptoms in *A. thaliana* plants associated with phytoplasma infection. Considering that phyllody symptoms are often observed in angiosperms infected by phytoplasmas, it is interesting to verify whether phyllogen could induce phyllody in diverse plant species by mediating degradation of MTFs, as is the case in *A. thaliana*.

In this study, phyllogen was expressed in several plant species known to show phyllody symptoms in response to phytoplasma infection; petunia (Himeno *et al.*, 2011; Faghihi *et al.*, 2014), sunflower (Guzmán *et al.*, 2014), aster (Sinha and Paliwal, 1969), and sesame (Akhtar *et al.*, 2009). Furthermore, it was examined whether phyllogen targets A- and E-class MTFs and their homologous MTFs from diverse plant species. The results indicate that phyllogen can inhibit MTF function in angiosperms and, surprisingly, also in gymnosperms and ferns, functioning as a broad-spectrum virulence factor manipulating floral morphology.

## Materials and methods

### Materials

Petunia (cv. Vakara White; Sakata Seed, http://www.sakata.com), *Nicotiana benthamiana*, sunflower (*Helianthus annuus* cv. Big Smile; Takii Seed, http://www.takii.co.jp), China aster (*Callistephus chinensis* cv. Nene White Imp; Takii Seed), and sesame (*Sesamum indicum* cv. Gomao; Takii Seed) were used. The plants were grown from seed under natural light conditions at 25 °C.

Among the MTF genes shown in Table 1, *SEP3* was cloned in a previous study (Maejima *et al.*, 2014*a*), and the ORFs of the other MTF genes used in the yeast two-hybrid (Y2H) and yellow fluorescent protein (YFP) reporter assays were synthesized by Thermo Fisher Scientific (https://www.thermofisher.com). The synthesized sequences lacked stop codons, but had additional sequences: 5'-AAGGAACCAATTCAGTC-3' at their 5' end and 5'-TATCTAGACCCAGCTT-3' at their 3' end. The synthesized *LMADS3* and *CRM6* gene sequences additionally lacked the codons for several N-terminal amino acids, because the corresponding nucleotide sequence was not available in GenBank (https://www.ncbi.nlm.nih.gov/genbank/). The *LMADS6* gene was synthesized with codon optimization because of the difficulty of synthesis (Supplementary Fig. S1A at *JXB* online).

**Table 1.** Plant genes used in this study

Gene	Plant	Family	Division	Class/function	Purpose	Reference	Accession no.
*SEP3*	*A. thaliana*	Brassicaceae	Angiospermae (eudicot)	E	Y2H, reporter assay	Pelaz *et al.* (2000)	NM_102272
*PFG*	*P. hybrida*	Solanaceae	Angiospermae (eudicot)	A^*a*^	Y2H, YFP reporter assay, qRT-PCR	Immink *et al.* (1999)	AF176782
*FBP29*	*P. hybrida*	Solanaceae	Angiospermae (eudicot)	A^*a*^	qRT-PCR	Immink *et al.* (2003)	AF335245
*PhAP2A* ^*b*^	*P. hybrida*	Solanaceae	Angiospermae (eudicot)	A^*a*^	qRT-PCR	Maes *et al.* (2001)	AF132001
*GLO1*	*P. hybrida*	Solanaceae	Angiospermae (eudicot)	B	qRT-PCR	Vandenbussche *et al.* (2004)	AY532265
*DEF*	*P. hybrida*	Solanaceae	Angiospermae (eudicot)	B	qRT-PCR	van der Krol *et al.* (1993)	DQ539416
*FBP6*	*P. hybrida*	Solanaceae	Angiospermae (eudicot)	C	qRT-PCR	Kater *et al.* (1998)	X68675
*pMADS3*	*P. hybrida*	Solanaceae	Angiospermae (eudicot)	C	qRT-PCR	Tsuchimoto *et al.* (1993)	X72912
*FBP2*	*P. hybrida*	Solanaceae	Angiospermae (eudicot)	E	Y2H, YFP reporter assay, qRT-PCR	Ferrario *et al.* (2003)	M91666
*FBP5*	*P. hybrida*	Solanaceae	Angiospermae (eudicot)	E	qRT-PCR	Immink *et al.* (2002)	AF335235
*FBP13*	*P. hybrida*	Solanaceae	Angiospermae (eudicot)	Flowering time gene^*c*^	qRT-PCR	Immink *et al.* (2003)	AF335237
*FBP25*	*P. hybrida*	Solanaceae	Angiospermae (eudicot)	Flowering time gene^*c*^	qRT-PCR	Immink *et al.* (2003)	AF335243
*UNS*	*P. hybrida*	Solanaceae	Angiospermae (eudicot)	Flowering time gene	qRT-PCR	Ferrario *et al.* (2004)	AF335238
*CDM111*	*C. morifolium*	Asteraceae	Angiospermae (eudicot)	A	Y2H, reporter assay	Shchennikova *et al.* (2004)	AY173054
*CDM44*	*C. morifolium*	Asteraceae	Angiospermae (eudicot)	E	Y2H, reporter assay	Shchennikova *et al.* (2004)	AY173057
*OsMADS14*	*O. sativa*	Poaceae	Angiospermae (monocot)	A^*a*^	Y2H, reporter assay	Jeon *et al.* (2000)	AF058697
*OsMADS8*	*O. sativa*	Poaceae	Angiospermae (monocot)	E	Y2H, reporter assay	Cui *et al.* (2010)	U78892
*LMADS6* ^*d*^	*L. longiflorum*	Liliaceae	Angiospermae (monocot)	A	Y2H, reporter assay	Chen *et al.* (2008)	HQ149332
*LMADS3* ^*e*^	*L. longiflorum*	Liliaceae	Angiospermae (monocot)	E^*f*^	Y2H, reporter assay	Tzeng *et al.* (2003)	AY826062
*CjMADS14*	*C. japonica*	Cupressaceae	Gymnospermae	Unknown	Y2H, reporter assay	Futamura *et al.* (2008)	AB359029
*DAL1*	*P. abies*	Pinaceae	Gymnospermae	Unknown	Y2H, reporter assay	Carlsbecker *et al.* (2004)	X80902
*CRM6* ^*e*^	*C. pteridoides*	Pteridaceae	Pteridophyta	Unknown	Y2H, reporter assay	Münster *et al.* (1997)	Y08242

^*a*^ Genes belonging phyllogenetically to A-class, but with undetermined functions.

^*b*^ Gene encodes a non-MTF protein.

^*c*^ Putative homolog of *AGL24*, a flowering time gene of *A. thaliana*, but with undetermined functions.

^*d*^ Gene was used with codon optimization.

^*e*^ Partial gene sequence was used.

^*f*^ Gene belonging phyllogenetically to E-class, but with undetermined functions.

Two phyllogens were used in this study: PHYL1_OY_ and PHYL1_PnWB_. PHYL1_PnWB_ was isolated from peanut witches’ broom (PnWB) phytoplasma (Chung *et al.*, 2013) and induced phyllody in *A. thaliana* (Yang *et al.*, 2015). The *PHYL1*_*OY*_ gene was cloned in a previous study (Maejima *et al.*, 2014*a*). A plant codon-optimized *PHYL1*_*PnWB*_ gene was also synthesized, as well as the MTFs, with a stop codon (Supplementary Fig. S1B). The synthesized genes were cloned into the pENTA vector (Himeno *et al.*, 2010) digested by *Sal*I-HF and *Eco*RV-HF (NEB; https://www.neb.com), using the GeneArt Seamless Cloning and Assembly Kit (Invitrogen; http://www.thermofisher.com), according to the manufacturer’s instructions.

### Virus vector construction

To express phyllogen in a wide range of plants, each of the phyllogens were cloned into the *Apple latent spherical virus* (ALSV) vector system, which can express foreign proteins in many plant species (Li *et al.*, 2004; Yamagishi *et al.*, 2011). DNA fragments harboring the 35S promoter and viral cDNAs of pEALSR1 and pEALSR2L5R5 (Li *et al.*, 2004) were cloned into the *Eco*RI and *Pma*CI sites of pCAMBIA1301, an *Agrobacterium* binary vector, using the GeneArt Seamless Cloning and Assembly Kit (pCAM-ALSV1 and pCAM-ALSR2L5R5, respectively). The multiple cloning sites (MCSs) of ALSV-RNA2 in pCAM-ALSR2L5R5 (*Xho*I, *Sma*I, and *Bam*HI) were replaced with 5'-GTCGACCCTAGGAGCGCTGGATCC-3' (*Sal*I, *Bln*I, *Aor*51HI, and *Bam*HI; designated pCAM-ALSV2), because two *Xho*I sites and one *Sma*I site were also present outside of the MCS of the pCAM-ALSR2L5R5 vector.

To construct pCAM-ALSV2, the new MCS was added at one end of an amplicon by a PCR, using 1301ALSV-newRest-F and 1301ALSV-newRest-R primers (Supplementary Table S1) and the ALSV-RNA2 cDNA template. The amplicon was inserted into the *Sac*I and *Bam*HI sites of the pCAM-ALSR2L5R5 vector by replacing the corresponding region. Furthermore, synonymous nucleotide substitutions were introduced in the vicinity of the duplicated cleavage site, just before the MCS, as follows: 5'-TTGTTGGAGGGACAAGGACCAGACTTTACT-3' (mutated sites are underlined; designated pCAM-ALSV2opt), to improve the stability of foreign genes in the virus vector. To construct pCAM-ALSV2opt, the synthesized DNA fragment with synonymous nucleotide substitutions was added at one end of an amplicon by PCR, using 1301ALSV-newRest-F and ALSV-NewCodon-R primers (Supplementary Table S1), and the ALSV-RNA2 cDNA template. The amplicon was inserted into the *Sac*I and *Sal*I sites of pCAM-ALSV2 by replacing the corresponding region. *PHYL1*_*OY*_ and *PHYL1*_*PnWB*_ genes were inserted into the *Sal*I and *Bam*HI sites of pCAM-ALSV2opt. pCAM-ALSV1 and pCAM-ALSV2opt, carrying no gene or either of the *PHYL1* genes, were used to transform the *Agrobacterium tumefaciens* strain EHA105.

### ALSV infiltration and detection

*Agrobacterium* cells containing ALSV-RNA1, each of the ALSV-RNA2 constructs, and a viral silencing suppressor P19, were adjusted to an optical density at 600 nm (OD_600_) of 1.0, and mixed at a ratio of 10:10:1, as described previously (Senshu *et al.*, 2009). They were co-infiltrated into 3-week-old petunia and *N. benthamiana* leaves, 2-week-old sunflower leaves, and 4-week-old China aster and sesame leaves. Inoculated plants were grown under a constant photoperiod (18 h light/6 h dark) at 25 °C.

Virus infection and foreign gene retention were confirmed by a two-step reverse transcription–PCR (RT–PCR). Total RNA was extracted from plants ~30 d after inoculation, using the cetyltrimethylammonium bromide (CTAB) method (Chang *et al.*, 1993) with minor modifications. Reverse transcription was performed as described previously (Minato *et al.*, 2014*b*). PCR was performed using KOD FX (Toyobo, http://www.toyobo.co.jp) and the primers 2600F and 3000R (Supplementary Table S1), which amplify a part of ALSV-RNA2, including the MCS region.

### Y2H assay

The Matchmaker GAL4 Two-Hybrid System 3 kit (Clontech; http://www.clontech.com) was used for Y2H assays, as described previously (Yamaji *et al.*, 2006). The BD-fused PHYL1_OY_ and AD-fused SEP3 were constructed as previously reported (Maejima *et al.*, 2014*a*). For construction of the other AD-fused MTFs, the MTFs were amplified using the primers shown in Supplementary Table S1, and cloned into the pGADT7 vector (Clontech) digested by *Nde*I and *Eco*RI-HF (NEB), as described above. The BD-fused PHYL1_PnWB_ was constructed in the same manner, using the pGBKT7 vector (Clontech), digested by *Nde*I and *Eco*RI-HF.

Lithium acetate-treated yeast cells (strain AH109) were co-transformed with pairs of appropriate pGADT7 and pGBKT7 vectors. Successful co-transformants were selected on synthetically defined medium (SD) lacking tryptophan and leucine (SD/–LW). To evaluate the protein interaction, the co-transformants were cultured on three selective media: leucine/tryptophan/histidine-lacking SD (SD/–LWH), SD/–LWH containing 5 mM 3-amino-1,2,4-triazole (Sigma-Aldrich) (SD/–LWH+3AT), and leucine/tryptophan/adenine/histidine-lacking SD (SD/–LWAH).

### Fluorescence microscopy

Constructs for the *in planta* expression of PHYL1_OY_, β-glucuronidase (GUS), YFP-fused bZIP63, or YFP-fused SEP3 were produced in a previous study (Maejima *et al.*, 2014*a*). Constructs for the expression of the other YFP-fused MTFs were obtained from pENTA carrying the MTFs and pEarleyGate101 binary vector (Earley *et al.*, 2006), using Gateway technology (Invitrogen). Protein expression, involving fluorescence observation in *N. benthamiana* leaves, was performed as described previously (Maejima *et al.*, 2014*a*). The number of fluorescent nuclei in a leaf area of 2.4 mm^2^ was quantified using Image J ver. 10.2 software (http://rsb.info.nih.gov/ij). The experiments were repeated independently three times.

### Protein extraction and immunoblotting

A construct for transient expression of PHYL1_PnWB_ was produced from pENTA carrying PHYL1_PnWB_ and the pFAST-G02 binary vector (Shimada *et al.*, 2010), using Gateway technology. *Agrobacterium* cells carrying SEP3–YFP and either PHYL1_PnWB_ or GUS constructs were co-infiltrated at a ratio of 1:1 into *N. benthamiana* leaves (4 weeks old) as described above. The leaves were collected 36 h after infiltration and pulverized in liquid nitrogen. Total protein was extracted with SDS sample buffer containing 5% 3-mercapto-1,2-propanediol. The extract was centrifuged at 15 000 rpm for 15 min at 4 °C to remove cell debris, and the supernatant was incubated for 5 min at 95 °C. The proteins were separated by SDS–PAGE, blotted onto a polyvinylidene difluoride membrane, and detected using an anti-green fluorescent protein (GFP) antibody (#1814460, Roche; http://www.roche.com). An immunoblotting assay of PHYL1_OY_ was performed using *Agrobacterium* cells carrying YFP-fused proteins and either PHYL1_OY_ or GUS constructs mixed in a ratio of 1:10. The experiments were repeated independently twice.

### Quantitative real-time PCR (qRT-PCR)

Total RNA was extracted from buds (<5 mm in diameter) of ALSV-PHYL1_OY_- or ALSV-empty-infected petunia plants, after confirming flower malformations in the ALSV-PHYL1_OY_-infected plants. Nine plants per ALSV construct were used. RNA extraction, purification, and reverse transcription were performed according to a previous report (Himeno *et al.*, 2011). A total of 500 ng of total RNA was used for reverse transcription. A qRT-PCR assay was performed using the Thermal Cycler Dice real-time PCR system (TaKaRa; http://www.takara-bio.co.jp) with SYBR Premix ExTaq (TaKaRa). A 2 µL aliquot of each 10-fold diluted cDNA was used as a template. The glyceraldehyde-3-phosphate dehydrogenase (*GAPDH*) gene was used for normalization, according to a previous report (Rijpkema *et al.*, 2006*b*; Himeno *et al.*, 2011). The primers used for qRT-PCR are shown in Supplementary Table S1. The PCR was repeated three times for each gene.

## Results

### Induction of phyllody phenotypes by PHYL1_OY_ and PHYL1_PnWB_ in solanaceous plants

**Fig. 1.**
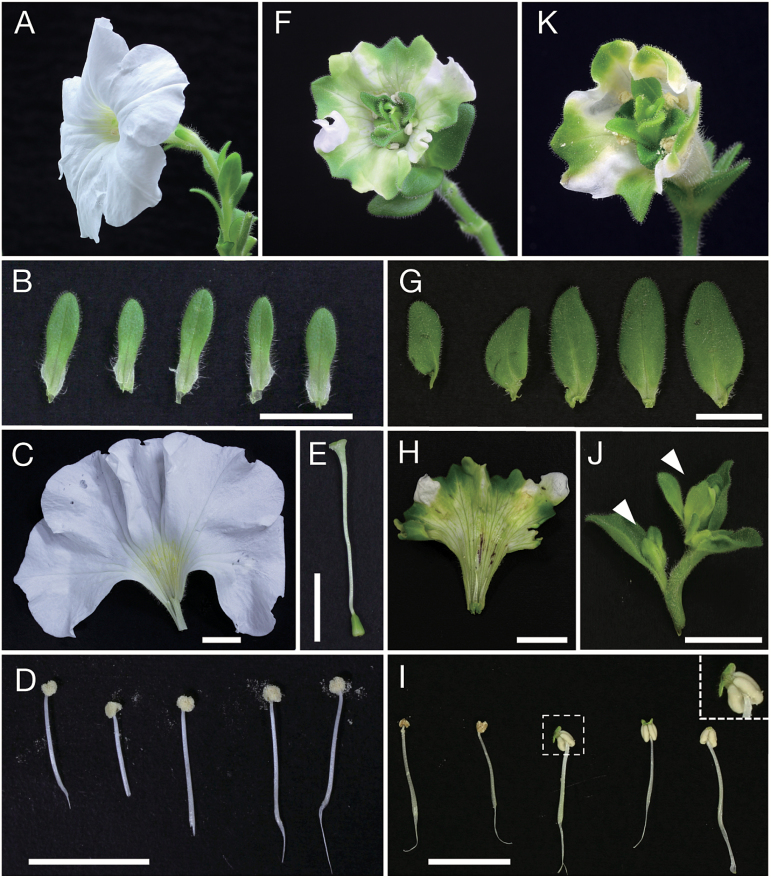
Characteristics of phyllogen-induced phyllody in petunia flowers. Whole flower (A) and each of the floral organs (B–E) of ALSV-empty-infected plants: (B) sepals, (C) petals, (D) stamens, and (E) a pistil. No malformation was observed in any floral organ. Whole flower (F) and each of the floral organs (G–J) of ALSV-PHYL1_OY_-infected plants. Flower malformations were observed in each floral organ. (G) Sepals showing leaf-like round shapes. (H) Petals becoming small and green at the tips and edges of the limbs. (I) Stamens with no pollen, but with small leaf-like structures on top of their anthers. Two left stamens did not show malformation, but pollen was already shed. An enlarged view of an abnormal anther (dotted square) is shown in the upper right of the picture. (J) Pistil replaced by leaf-like structures with trichomes. Arrowheads indicate newly developed floral buds. (K) Flower of ALSV-PHYL1_PnWB_-infected petunia. The observed phyllody was very similar to that of ALSV-PHYL1_OY_-infected petunia plants. Bars indicate 1 cm.

Two phyllogens (PHYL1_OY_ and PHYL1_PnWB_) were initially expressed in petunia, a model solanaceous plant for genetic studies of floral organ development. Before the experiment, it was confirmed that PHYL1_PnWB_ interacted with SEP3 in yeast cells and induced its degradation *in planta* (Supplementary Fig. S2). Each phyllogen was expressed in petunia plants using ALSV. The constructed vectors (ALSV-PHYL1_OY_ and ALSV-PHYL1_PnWB_) and the ALSV-empty vector were introduced into *Agrobacterium* and inoculated into petunia plants. Successful infection of these plants was confirmed by RT–PCR (data not shown). At 40–60 d after inoculation, petunia plants infected with ALSV-PHYL1_OY_ showed phyllody phenotypes (Fig. 1F–J), while those infected with the ALSV-empty vector produced normal flowers (Fig. 1A–E). All floral organs in ALSV-PHYL1_OY_-infected petunia plants were affected. Sepals became large, and were converted into a leaf-like, round shape (Fig. 1G). Petals became green, especially at the tips and edges of the limbs, and their size was reduced (Fig. 1H). Stamens occasionally had no pollen, and small leaf-like structures were formed on top of the anthers (Fig. 1I). In the development of pistils, leaf-like structures with trichomes replaced normal ovules, and new inflorescences with floral buds developed (Fig. 1J). ALSV-PHYL1_PnWB_ also caused phyllody phenotypes in petunia, in a similar manner to ALSV-PHYL1_OY_ (Fig. 1K).

PHYL1_OY_ and PHYL1_PnWB_ also affected flower morphology in another solanaceous plant, *N. benthamiana* (Supplementary Fig. S3). In ALSV-PHYL1_OY_-infected *N. benthamiana*, the sepals were initially enlarged (Supplementary Fig. S3B), and the pistils turned into leaf-like structures (Supplementary Fig. S3C). Then the flowers developed small, greenish, and fused petals (Supplementary Fig. S3D). In later flowers, all floral organs were converted into leaf-like structures as in phyllody (Supplementary Fig. S3E). ALSV-PHYL1_PnWB_ caused flower malformations very similar to those caused by ALSV-PHYL1_OY_ (Supplementary Fig. S3F).

### PHYL1_PnWB_ induces phyllody in various plant species

**Fig. 2.**
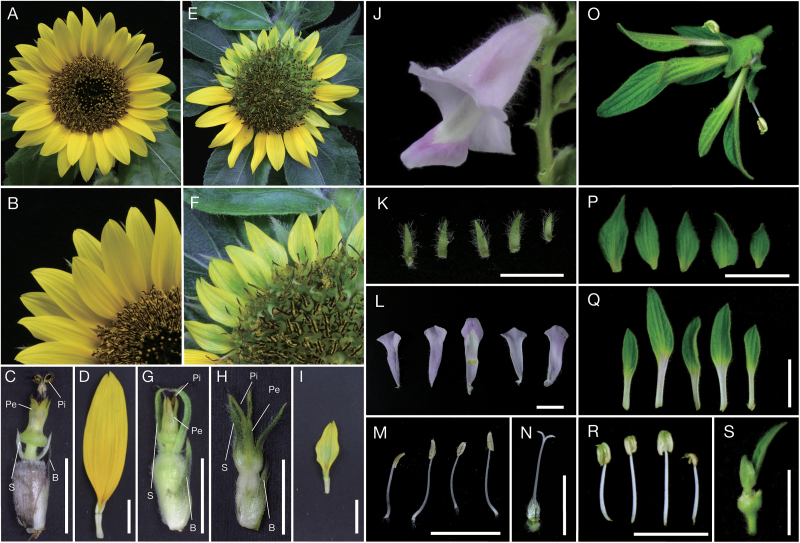
Characteristics of PHYL1_PnWB_-induced phyllody on sunflower and sesame flowers. (A–D) Flowers of ALSV-empty-infected sunflower. A disc floret and ray floret are shown in (C) and (D), respectively. No flower malformation was observed in any floral organ. (E–I) Flowers of ALSV-PHYL1_PnWB_-infected sunflower. Flower malformations were observed on both disk and ray florets. (G) Disk floret showing mild malformations. Bract and sepals became green and elongated. (H) Disk floret showing severe malformations. Petals became green and a pistil changed into a leaf-like structure. (I) Ray floret showing malformations. Corolla became small, and its color changed slightly to green. (J–S) Characteristics of phyllogen-induced phyllody in sesame flowers. Whole flower (J) and each of the floral organs (K–N) of ALSV-empty-infected sesame plants: (K) sepals, (L) separated petals, (M) stamens, and (N) a pistil. No malformation was observed in any floral organ. Whole flower (O) and each of the floral organs (P–S) of ALSV-PHYL1_PnWB_-infected plants. Flower malformations were observed in each floral organ. (P) Sepals showing large leaf-like structures. (Q) Petals becoming green from the tips. (R) Stamens with no pollen, but with small leaf-like structures on top of their anthers. (S) Pistils replaced by leaf-like structures. Bars indicate 1 cm. B, bracts; S, sepals; Pe, petals; Pi, pistils.

ALSV-PHYL1 was inoculated to sunflower (family Asteraceae), China aster (family Asteraceae), and sesame (family Pedaliaceae). Because the inserted sequence of the PHYL1_OY_ gene was less stable in the ALSV viral vector compared with that of PHYL1_PnWB_ in sunflower (data not shown), ALSV-PHYL1_PnWB_ was used in this experiment. While ALSV-empty-infected sunflowers developed normal flowers (Fig. 2A–D), ALSV-PHYL1_PnWB_-infected sunflowers showed flower malformations (Fig. 2E–I). Specifically, bracts and sepals of disc florets were changed into green organs, and sepals were elongated (Fig. 2G). In severely affected disc florets, petals became green, and pistils changed into leaf-like structures (Fig. 2H). In ray florets, the size of the corolla was occasionally reduced and the color changed slightly to green (Fig. 2I). ALSV-PHYL1_PnWB_-infected China asters also exhibited similar flower malformations (Supplementary Fig. S4).

PHYL1_PnWB_ expression resulted in phyllody phenotypes in all floral organs of sesame (Fig. 2J–S), in a manner very similar to that observed in petunia. Sepals were converted into large leaf-like structures (Fig. 2P), petals became green from the tips (Fig. 2Q), stamens occasionally had no pollen and also became green from the tops of the anthers (Fig. 2R), and pistils were replaced by leaf-like structures (Fig. 2S).

### PHYL1_OY_ targets A- and E-class MTFs of various plant species

**Fig. 3.**
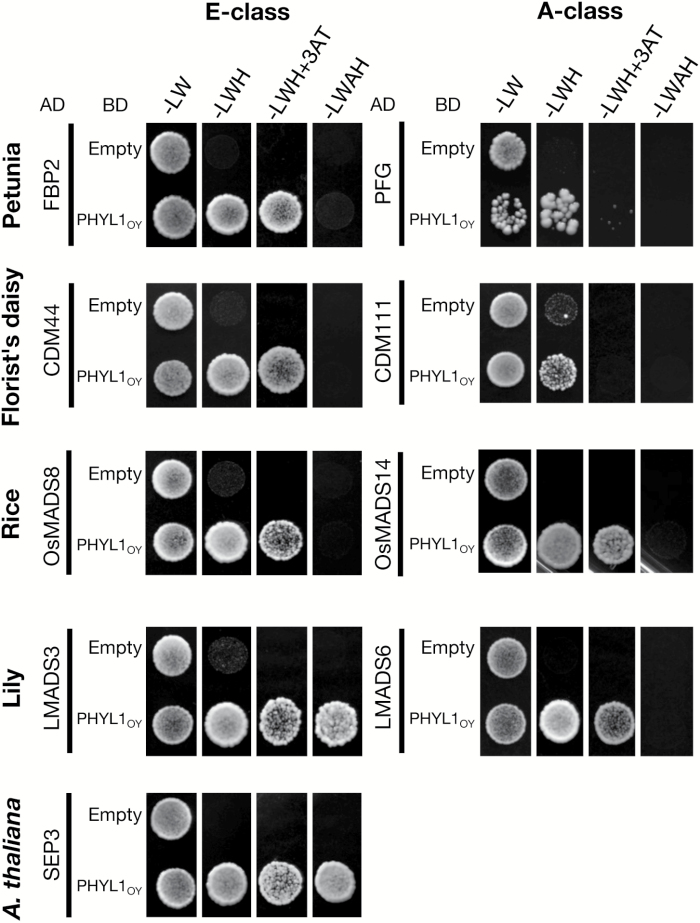
PHYL1_OY_ interacts with A- and E-class MADS domain transcription factors (MTFs) of angiosperms in yeast cells. The MTFs listed in Table 1 were fused to the GAL4 activation domain (AD). These AD-fused MTFs were expressed in the yeast strain AH109, with the GAL4 DNA-binding domain (BD) or BD-fused PHYL1_OY_. The AD-fused SEP3 is a positive control, which interacts with PHYL1_OY_ (Maejima *et al.*, 2014*a*). Yeast cells harboring the appropriate AD and BD vectors were adjusted to an OD_600_ of 0.1. Aliquots (10 µl) of these cells were spotted on synthetically defined medium lacking leucine/tryptophan (–LW), lacking leucine/tryptophan/histidine (–LWH), lacking leucine/tryptophan/histidine and containing 5 mM 3-amino-1,2,4-triazole (–LWH+3AT), or lacking tryptophan/leucine/adenine/histidine (–LWAH). The plates were incubated for 4 d at 30 °C.

To investigate whether PHYL1_OY_ interacts with A- and E-class MTFs of diverse plant species and leads to their degradation, as in the case of *A. thaliana*, the activities of PHYL1_OY_ against the MTFs from several angiosperms (Table 1) were examined. These MTFs were selected not only from the two eudicot species petunia (family Solanaceae) and florist’s daisy (*Chrysanthemum morifolium*, family Asteraceae), but also from the monocot species rice (*Oryza sativa*, family Poaceae) and lily (*Lilium longiflorum*, family Liliaceae). SEP3 of *A. thaliana* was used as a positive control. Yeast cells expressing BD-fused PHYL1_OY_ (BD-PHYL1_OY_), and each of the AD-fused MTFs, grew on selective media, while no growth was observed in yeast expressing empty BD and the AD-fused MTFs (Fig. 3).

**Fig. 4.**
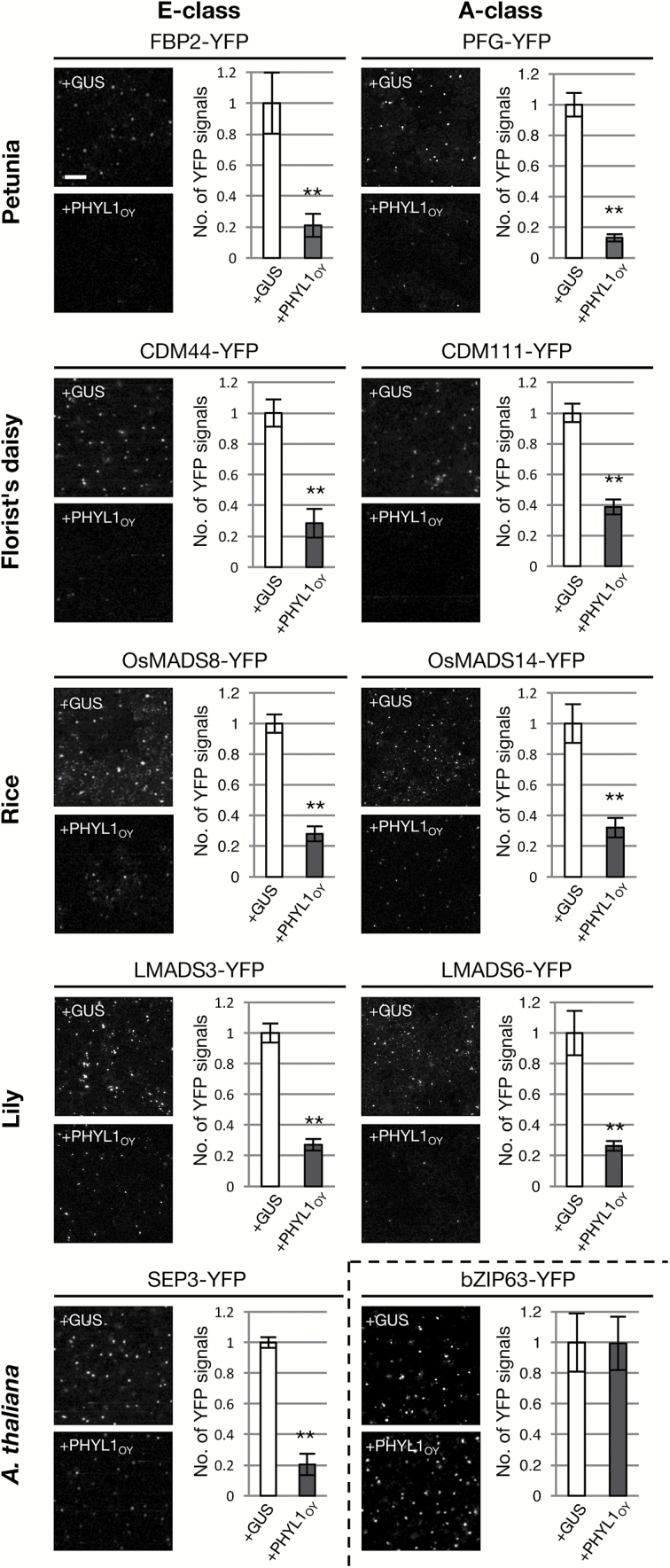
PHYL1_OY_ induces degradation of A- and E-class MADS domain transcription factors (MTFs) of angiosperms in *Nicotiana benthamiana* epidermal cells. Each of the MTFs was fused to the YFP protein and co-expressed with either GUS or PHYL1_OY_. We used bZIP63–YFP and SEP3–YFP as negative and positive controls, respectively. *Agrobacterium* cultures (OD_600_=1.0) carrying each of the YFP-fused proteins and those carrying either GUS or PHYL1_OY_ were mixed at a ratio of 1:10, and infiltrated into *N. benthamiana* leaves. YFP fluorescence was observed 36 h after infiltration. Graphs show the number of the YFP signals quantified in a relative manner. The average YFP signal of each MTF expressed with GUS was set as 1.0. Each bar represents the average of YFP signals observed in four leaf areas of 2.4 mm^2^. The bar indicates 100 µm. Asterisks indicate statistically significant differences compared with GUS (**P<0.01 by the one-tailed Student’s *t*-test). The experiment was performed independently three times. (This figure is available in colour at *JXB* online.)

Next, to test whether the MTFs were degraded in the presence of PHYL1_OY_*in planta*, each of the YFP-fused MTFs was expressed transiently with PHYL1_OY_ or GUS in *N. benthamiana* leaves by agro-infiltration. When GUS was co-expressed, YFP fluorescence signals were observed, mainly in the cell nuclei (Fig. 4). On the other hand, the YFP fluorescence signals were significantly reduced upon co-expression with PHYL1_OY_ (Fig. 4), showing that PHYL1_OY_ induced degradation of all of the MTFs used in this experiment. Co-expression of PHYL1_OY_ did not affect the accumulation and subcellular localization of YFP-fused bZIP63, a nuclear-localized leucine zipper transcription factor (control).

### PHYL1_OY_ represses MTF gene expression in petunia

**Fig. 5.**
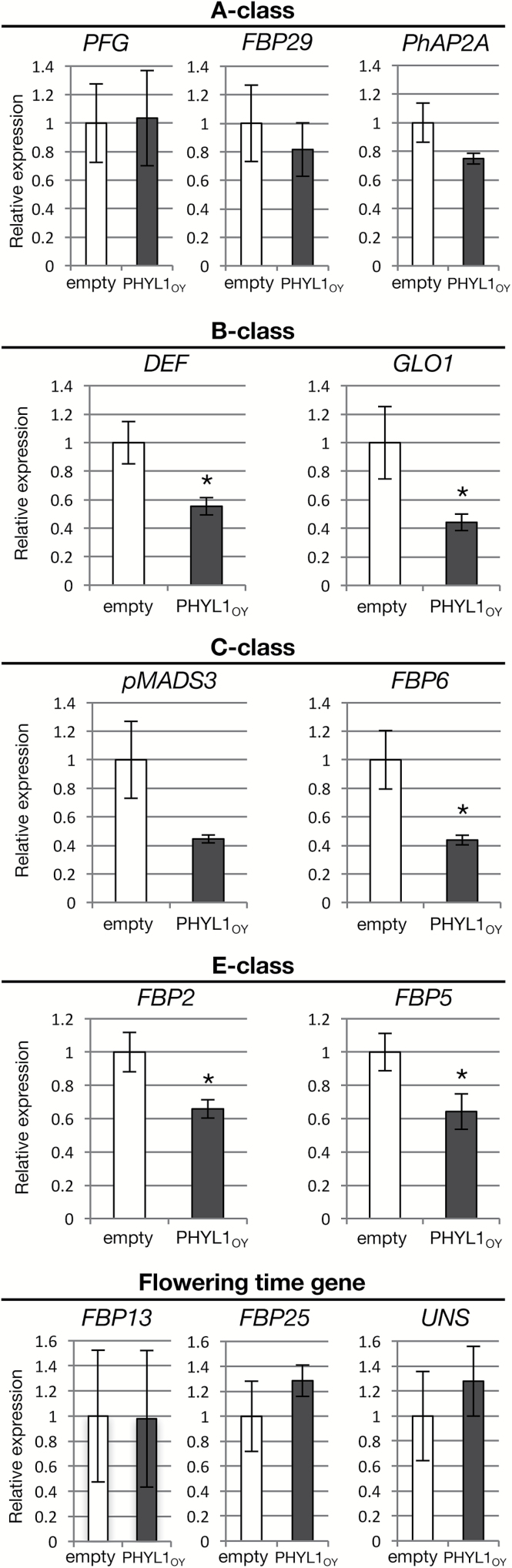
qRT-PCR analyses of MADS domain transcription factor (MTF) genes in floral buds of ALSV-empty- and ALSV-PHYL1_OY_-infected plants. Each bar represents the average of nine plants. The expression levels of the glyceraldehyde-3-phosphate dehydrogenase gene (*GAPDH*) were used for normalization. The average expression levels in the control were set as 1.0. Asterisks indicate statistically significant differences compared with the control (*P<0.05 by the two-tailed Student’s *t*-test).

To investigate the indirect effects of phyllogen on the MTFs, the expression levels of ABCE-class genes and flowering time genes was examined in floral buds of petunia plants affected by PHYL1_OY_. The expression of MTF genes shown in Table 1 in ALSV-PHYL1_OY_-infected plants was analyzed by qRT-PCR and compared with that in ALSV-empty-infected plants (Fig. 5). The A-class genes (*PFG*, *FBP29*, and *PhAP2A*) were not affected by PHYL1_OY_. On the other hand, the B- (*DEF* and *GLO1*), C- (*pMADS3* and *FBP6*), and E- (*FBP2* and *FBP5*) class genes were down-regulated in ALSV-PHYL1_OY_-infected plants, although the differences were not statistically significant for *pMADS3* (*P*=0.0573). The flowering time genes (*UNS*, *FBP13*, and *FBP25*) were not affected by PHYL1_OY_ (Fig. 5). These results show that PHYL1_OY_ affects the expression levels of the B-, C-, and E-class MTF genes in floral buds of petunia. Considering the induction of degradation of the A- and E-class MTF proteins (Fig. 4), PHYL1_OY_ has negative effects on the expression of all of the ABCE-class MTFs in petunia at the mRNA or protein levels.

### PHYL1_OY_ targets MTFs of gymnosperms and a fern

**Fig. 6.**
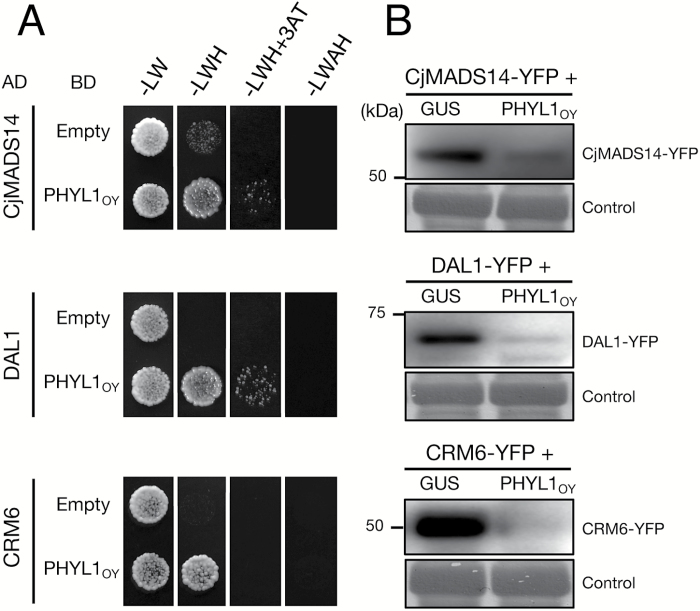
PHYL1_OY_ targets the MTFs of gymnosperms and a fern. MADS domain transcription factors (MTFs) were chosen from two gymnosperms (CjMADS14 from *Cryptomeria japonica* and DAL1 from *Picea abies*) and a fern (CRM6 from *Ceratopteris pteridoides*). (A) A yeast two-hybrid assay showed interaction between PHYL1_OY_ and the MTFs in yeast cells. Each of the AD-fused MTFs was expressed in the yeast strain AH109, with BD or BD-fused PHYL1_OY_. Experimental details are described in the legend to Fig. 3. (B) The YFP reporter assay showed PHYL1_OY_-dependent degradation of the MTFs in *Nicotiana benthamiana* leaves. Accumulation of each MTF was evaluated by immunoblotting using an anti-GFP antibody.

The interactions between PHYL1_OY_ and the MTFs of two gymnosperms (CjMADS14 of *Cryptomeria japonica* and DAL1 of *Picea abies*; Supplementary Fig. S5) and the MTF of a fern (CRM6 of *Ceratopteris pteridoides*; Supplementary Fig. S5) were investigated by a Y2H assay. Yeast cells expressing BD-PHYL1_OY_ and each of the AD-fused MTFs grew on selective media, while those expressing empty BD and the AD-fused MTFs did not (Fig. 6A). Furthermore, accumulation of each MTF in *N. benthamiana* leaves was evaluated using the YFP reporter assay, in combination with either the GUS protein or PHYL1_OY_. Because the subcellular localization of each of the YFP-fused MTFs was not limited to nuclei, the protein accumulation was confirmed by immunoblotting. Compared with GUS, co-expression with PHYL1_OY_ decreased the amount of these MTFs significantly (Fig. 6B). These results showed that PHYL1_OY_ interacted with, and induced degradation of these MTFs.

## Discussion

The function and molecular mechanism of phyllogen as a phyllody-inducing factor has been elucidated only in *A. thaliana,* a well-studied and easily transformed model plant (MacLean *et al.*, 2011; Maejima *et al.*, 2014*a*; Yang *et al.*, 2015). In this study, it was investigated whether phyllogen could induce phyllody phenotypes in various plants. Two phyllogens (PHYL1_OY_ and PHYL1_PnWB_) were initially expressed in petunia, a model plant for genetic studies of floral organ development (Gerats and Vandenbussche, 2005; Rijpkema *et al.*, 2006*a*). The results showed that PHYL1_OY_ and PHYL1_PnWB_ have phyllody-inducing activity in petunia (Fig. 1). They also induced phyllody and abnormal flower development in another solanaceous plant, *N. benthamiana* (Supplementary Fig. S3), indicating that PHYL1_OY_ and PHYL1_PnWB_ can induce phyllody in solanaceous plants. Moreover, PHYL1_PnWB_ induced phyllody in sunflower (family Asteraceae; Fig. 2), China aster (family Asteraceae; Supplementary Fig. S4), and sesame (family Pedaliaceae; Fig. 2) when it was expressed using ALSV vector. Reports from previous studies on phyllogen-induced phyllody phenotypes in *A. thaliana* (MacLean *et al.*, 2011; Maejima *et al.*, 2014*a*; Yang *et al.*, 2015) and the results from the current study indicate that phyllogen can induce phyllody phenotypes in eudicots, at least those belonging to the four families Brassicaceae, Solanaceae, Asteraceae, and Pedaliaceae. Furthermore, PHYL1_OY_ recognized and induced degradation of the A- and E-class MTFs of two eudicots (petunia and the florist’s daisy), and also those of two monocots (rice and lily; Figs 3, 4), as in *A. thaliana* (Maejima *et al.*, 2014*a*, 2015). This strongly suggests that PHYL1_OY_ and other phyllogens can recognize A- and E-class MTFs in diverse angiosperms. In *A. thaliana*, phyllogen induces degradation of target MTFs via a proteasome-mediated pathway (MacLean *et al.*, 2014; Maejima *et al.*, 2014*a*). Considering that the ABCE-class MTF genes and the proteasome-mediated protein degradation pathway are conserved in plants (Ingvardsen and Veierskov, 2001; Zahn *et al.*, 2005*a*; Kater *et al.*, 2006), our results strongly suggest that phyllogen is a broad-spectrum virulence factor that can repress the functions of A- and E-class MTFs in a large range of angiosperm species.

It will be interesting to investigate whether phyllogen-mediated degradation of the MTFs (Figs 3, 4) affects the floral morphology of monocots. In rice, RNA silencing of two E-class genes (OsMADS7 and OsMADS8) causes severe morphological alterations of floral organs, including their conversion into leaf-like organs (Cui *et al.*, 2010). In other monocot species, although information on loss-of-function mutants of A- and E-class genes is very limited, many reports suggest that A-class and E-class gene homologs are involved in floral organ development (Caporali *et al.*, 2000; Yu and Goh, 2000; Adam *et al.*, 2007; Acri-Nunes-Miranda and Mondragón-Palomino, 2014; Kubota and Kanno, 2015). Therefore, it is likely that phyllogen-mediated degradation of the A- and E-class MTFs induces phyllody or other flower malformations in monocots.

The phenotypes of PHYL1-affected petunia flowers were very similar to those of E-class knockdown or knockout petunia mutants, in terms of the development of size-reduced green corollas, transformation of stamens into green petaloid structures, and development of inflorescences in the axils of the carpels (Fig. 1; Angenent *et al.*, 1994; Vandenbussche *et al.*, 2003). In contrast, knockdown of A-class MTF genes in petunia does not produce leaf-like flower malformations (Immink *et al.*, 1999). Moreover, in the petunia buds expressing PHYL1_OY_, the B- and C-class genes were down-regulated (Fig. 5), which was similar to the expression pattern in E-class knockdown in petunia (Angenent *et al.*, 1994). These data suggest that downregulation of E-class MTFs, rather than A-class MTFs, contributes mainly to phyllogen-induced phyllody symptoms. This might be because E-class MTFs play an important role as mediators of every higher order complex formation in the floral quartet model (Smaczniak *et al.*, 2012). Considering the previous findings that phyllogen also induces phyllody phenotypes and loss of floral meristem determinacy like E-class mutants (Pelaz *et al.*, 2000; Ditta *et al.*, 2004), and degrades all E-class MTFs of *A. thaliana* (Maejima *et al.*, 2014*a*, 2015), it is reasonable to conclude that phyllody phenotypes induced by the expression of phyllogen are attributed largely to the degradation of E-class MTFs.

Gymnosperms and ferns have MTF genes phylogenetically related to MTFs of angiosperms (Theissen *et al.*, 2000; Becker and Theissen, 2003; Gramzow *et al.*, 2010; Gramzow *et al.*, 2014). This work has shown that, in addition to recognizing MTFs from angiosperms, PHYL1_OY_ recognized and induced degradation of MTFs from gymnosperms (CjMADS14 and DAL1; Fig. 6). CjMADS14 and DAL1 are assumed to belong to the AGL6-like protein family (Tandre *et al.*, 1995; Futamura *et al.*, 2008), being phylogenetically related to A- and E-class MTFs (Kim *et al.*, 2013). AGL6, whose homologs exhibit E-class functions in some angiosperms (Rijpkema *et al.*, 2009; Dreni and Zhang, 2016), has been shown to interact with a member of the phyllogen family, SAP54 (MacLean *et al.*, 2014). Our data and the cited reports suggest that phyllogens generally recognize these closely related MTFs. Furthermore, PHYL1_OY_ is shown to target CRM6, an MTF from a fern (Fig. 6). These data indicate that phyllogen may recognize a motif conserved in MTFs across the Plantae. MacLean *et al.* (2014) reported that SAP54 recognized the K-domains of MTFs of *A. thaliana.* Based on this report and the X-ray crystal structure of the K-domain of SEP3 (Puranik *et al.*, 2014), Rümpler *et al.*, (2015) suggested a hypothesized mode of interaction between SAP54 and MTFs. Our data will be useful to elucidate the conserved motif in the K-domain recognized by phyllogen, and to improve the hypothesis.

Broad-spectrum activity of phyllogen suggests that it can be used as a tool for engineering functions of MTFs. Because MTF repression could lead to unique and highly attractive flower malformations, MTFs are considered important targets for the genetic engineering of floral traits (Mol *et al.*, 1995; Matsubara *et al.*, 2008). RNA silencing is widely used to repress MTFs in many plant species (Angenent *et al.*, 1994; Cui *et al.*, 2010; Wang *et al.*, 2015). Because RNA silencing is a sequence-specific repression method (Brodersen and Voinnet, 2006; Martínez de Alba *et al.*, 2013), sequence information on target MTF genes is utilized for gene silencing in most studies (Aida *et al.*, 2008; Galimba *et al.*, 2012; Nakatsuka *et al.*, 2015). However, the floral MTF sequences have not yet been elucidated for many horticultural plants. This study suggests that phyllogen induces phyllody and other flower malformations in a wide range of angiosperms, even if the plant species are not well studied genetically (Fig. 2; Supplementary Figs S3, S4). Thus, phyllogen may be a useful alternative tool to engineer flower organs in a genetically dominant fashion. In addition, phyllogen is indicated to target MTFs of gymnosperms and ferns (Fig. 6). The functions of MTF genes in gymnosperms and ferns remain unclear, largely due to the lack of loss-of-function mutants. For example, ectopic expression analyses of *CjMADS14* or *DAL1* in *A. thaliana* suggest that these genes have been presumed to play a role during reproductive organ development (Carlsbecker *et al.*, 2004; Katahata *et al.*, 2014), but their actual functions in gymnosperms are not confirmed. This study suggests that phyllogen expression in gymnosperms and ferns would cause the inhibition of their MTF functions, which might be helpful to study their roles.

Considering that phytoplasmas infect hundreds of plant species, it appears that they have strategies to infect diverse host plants. Phytoplasmas are phloem-limited bacteria, and phyllody symptoms increase leaf-like organs where they can colonize (Arashida *et al.*, 2008; Su *et al.*, 2011). We found that phyllogen could induce phyllody in diverse plant species. This indicates that phyllogen creates favorable conditions for phytoplasma colonization in diverse angiosperms. Moreover, phytoplasmas can infect gymnosperms and cause symptoms such as stunting and needle malformations (Paltrinieri *et al.*, 1998; Schneider *et al.*, 2005; Davis *et al.*, 2010; Kamińska and Berniak, 2011). It would be interesting to study whether phyllogen is involved in these symptoms through the degradation of the MTFs, although further studies are needed to understand the functions of phyllogen and MTFs in gymnosperms. Additionally, a recent report suggested that SAP54 was involved in insect attraction, independent of phyllody induction, in *A. thaliana* (Orlovskis and Hogenhout, 2016). Given the importance of insect transmission to phytoplasmas, it will be interesting to explore whether phyllogen also enhances insect preferences in other plant species.

It is common for functionally analyzed phytoplasma virulence factors to target diverse plant species. To date, three virulence factors have been identified in phytoplasmas: phyllogen, TENGU (Hoshi *et al.*, 2009; Sugawara *et al.*, 2013; Minato *et al.*, 2014*a*), and SAP11 (Sugio *et al.*, 2011, 2014; Tan *et al.*, 2016; Janik *et al.*, 2017). Functions of each protein were tested in several plant species belonging to different families. Functions of phyllogen were tested in *A. thaliana*, petunia, *N. benthamiana*, sunflower, China aster, and sesame; functions of TENGU were tested in *A. thaliana* and *N. benthamiana*; and functions of SAP11 were tested in *A. thaliana* and *N. benthamiana*. SAP11 was also suggested to function in apple (Tan *et al.*, 2016). Virulence factors targeting diverse plants might be a common strategy for phytoplasmas to infect diverse plant species.

## Supplementary data

Supplementary data are available at *JXB* online.

Fig. S1. The optimized nucleotide sequences of *LMADS6* and *PHYL1*_*PnWB*_ used in the present study.

Fig. S2. PHYL1_PnWB_ interacts with SEP3 and induces its degradation.

Fig. S3. Characteristics of *Nicotiana benthamiana* flowers exhibiting phyllody.

Fig. S4. Characteristics of China aster flowers exhibiting phyllody.

Fig. S5. Unrooted phylogenetic tree of MTFs used in this study.

Table S1. Sequences of the primers used in this study.

## Supplementary Material

Supplementary_Figures_S1_S5_Table_S1
