# Supplementary material for: Phytoplasma-conserved phyllogen proteins induce phyllody across the Plantae by degrading floral MADS domain proteins
Source: J Exp Bot. 2017 May 15;68(11):2799–811. doi: 10.1093/jxb/erx158 (PMC5853863; doi:10.1093/jxb/erx158)
Supplement: Supplementary_Figures_S1_S5_Table_S1 [file erx158_suppl_supplementary_figures_s1_s5_table_s1.pdf]

Supplementary materials

Title
Phytoplasma-conserved phyllogen proteins induce phyllody across the Plantae by degrading floral MADS domain proteins

Authors
Yugo Kitazawa, Nozomu Iwabuchi, Misako Himeno, Momoka Sasano, Hiroaki Koinuma, Takamichi Nijo, Tatsuya Tomomitsu, Tetsuya Yoshida, Yukari Okano, Nobuyuki Yoshikawa, Kensaku Maejima, Kenro Oshima, and Shigetou Namba

Figure S1.
The optimized nucleotide sequences of *LMADS6* and *PHYL1*$_{PnWB}$ used in the present study.
Figure S2.
PHYL1$_{PnWB}$ interacts with SEP3 and induces its degradation.
Figure S3.
Characteristics of *Nicotiana benthamiana* flowers exhibiting phyllody.
Figure S4.
Characteristics of China aster flowers exhibiting phyllody.
Figure S5.
Unrooted phylogenetic tree of MTFs used in this study.
Table S1.
Sequences of the primers used in this study.

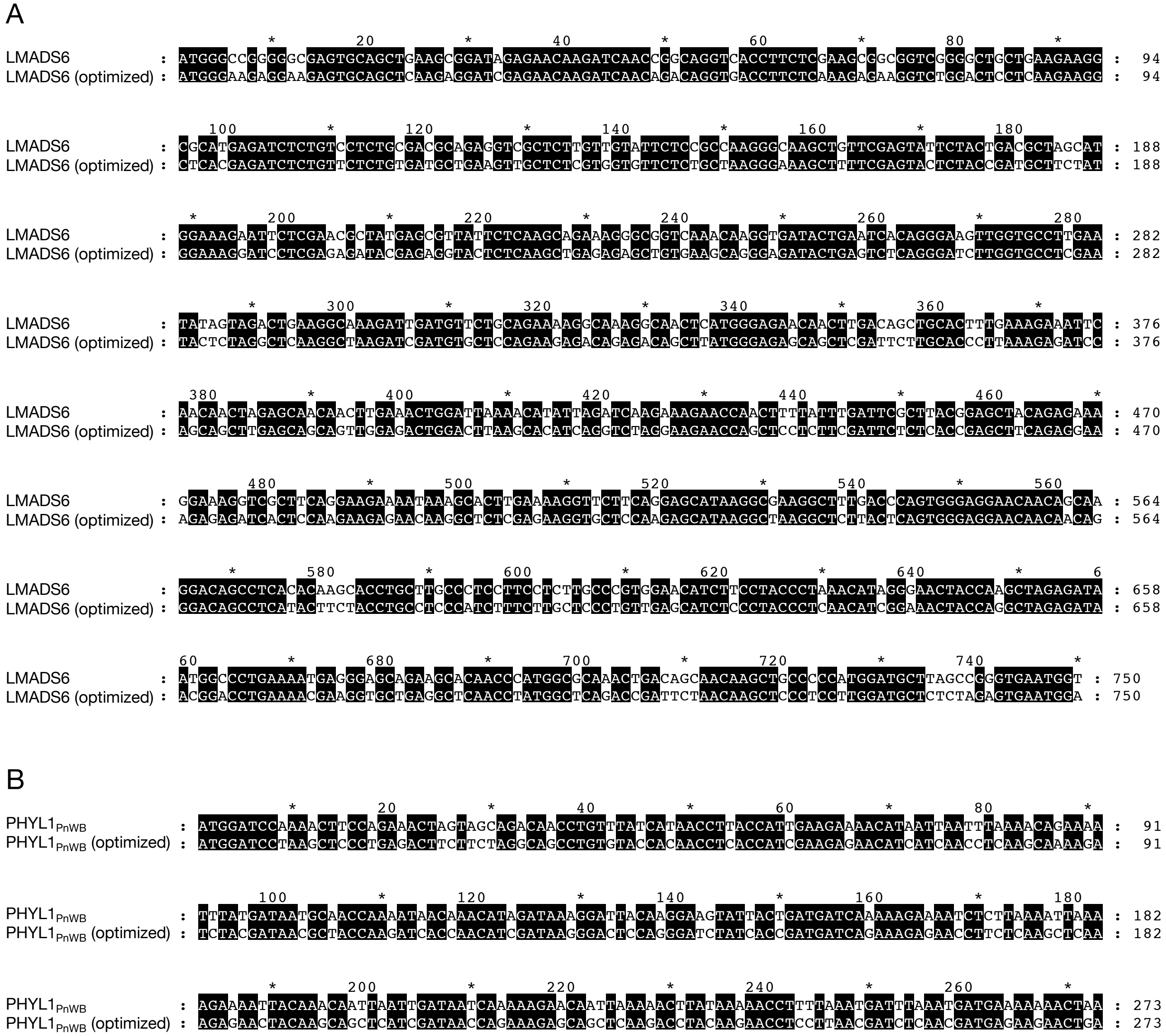

Supplementary Figure S1. The optimized nucleotide sequences of *LMADS6* (A) and *PHYL1$_{PnWB}$* (B) used in this study. Conserved nucleotides are indicated by black boxes. The stop codon of *LMADS6* is not shown.

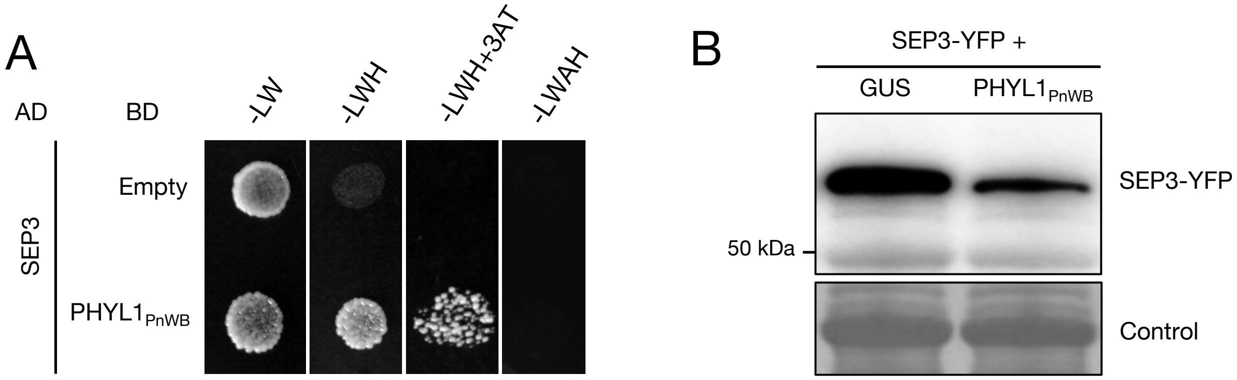

Supplementary Figure S2. PHYL1$_{PnWB}$ interacts with SEP3 and induces its degradation. (A) The yeast two-hybrid assay showed interaction between AD-SEP3 and BD or BD-PHYL1$_{PnWB}$ in yeast cells. Experimental details are described in the legend to Figure 4. (B) Accumulation of transiently expressed YFP-fused SEP3 (SEP3-YFP) in *Nicotiana benthamiana* leaves. *Agrobacterium* cultures (OD_{600} = 1.0) expressing SEP3-YFP, and either GUS or PHYL1$_{PnWB}$, were mixed at a ratio of 1:1, and infiltrated into *N. benthamiana* leaves. SEP3-YFP was detected 36 h after infiltration by immunoblotting using an anti-GFP antibody.

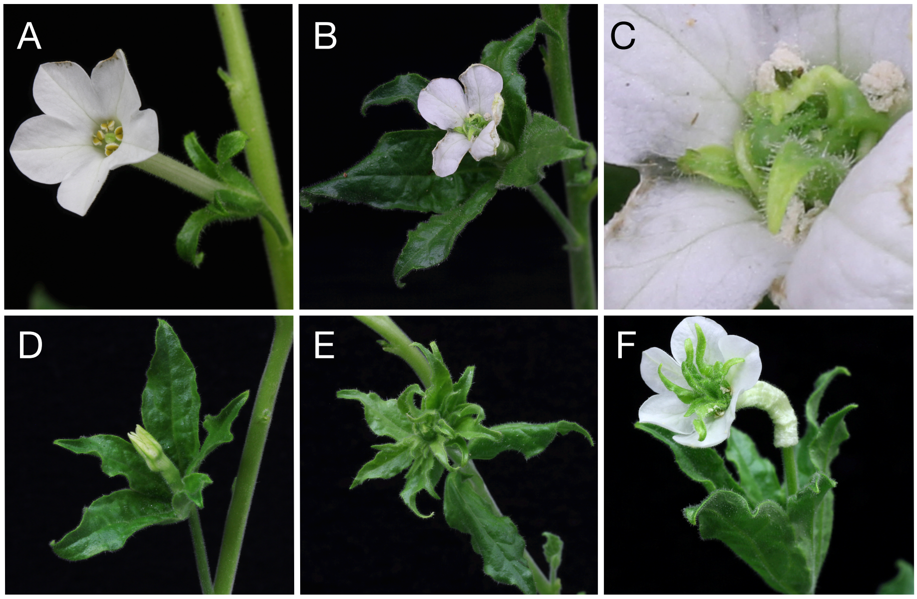

Supplementary Figure S3. Characteristics of *Nicotiana benthamiana* flowers exhibiting phyllody. (A) Flower of ALSV-empty-infected *N. benthamiana*. No malformation was observed in each floral organ. (B–E) Flowers of ALSV-PHYL1$_{OY}$-infected *N. benthamiana*. Flower showing mild malformation (B) and close-up view (C). Sepals were enlarged and a pistil turned into a leaf-like structure. (D) Flower with small and fused petals. (E) Flower showing phyllody in all floral organs. (F) Flower of ALSV-PHYL1$_{PnWB}$-infected *N. benthamiana*. The observed phyllody was very similar to that of ALSV-PHYL1$_{OY}$-infected plants.

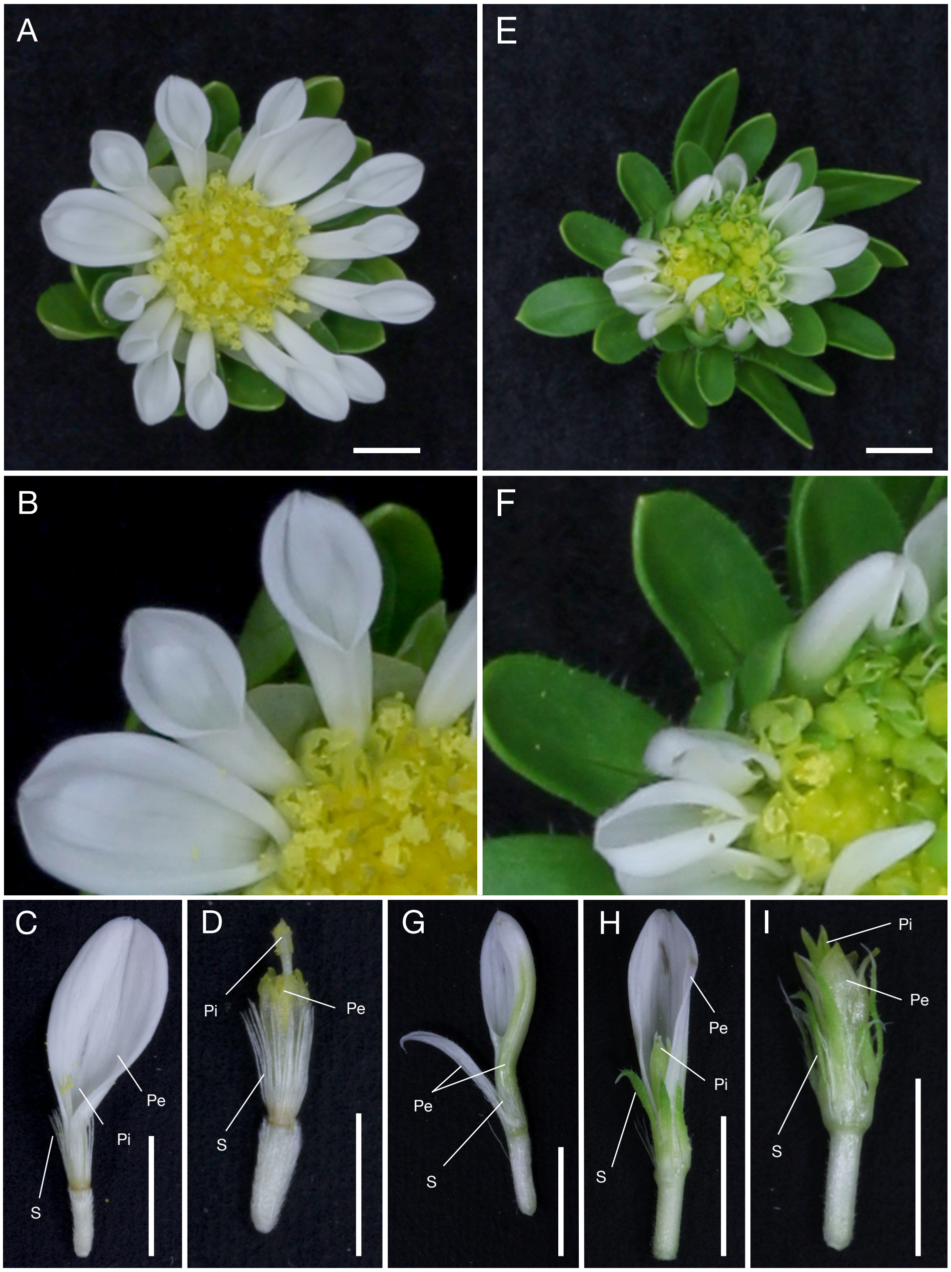

Supplementary Figure S4. Characteristics of China aster flowers exhibiting phyllody. (A–D) Flowers of ALSV-empty-infected China aster. A ray floret and a disk floret are shown in (C) and (D) respectively. No flower malformation was observed in any floral organ. (E–I) Flowers of ALSV-PHYL1$_{PnWB}$-infected China aster. Flower malformations were observed in both ray and disk florets. (G) A ray floret exhibiting mild malformation. The petal is slightly green and developed abnormally. (H) A ray floret exhibiting severe malformation. The partial sepals changed to leaf-like structures and became elongated. (I) A disk floret exhibiting malformations. Leaf-like elongated sepals are evident. A pistil also changed into a leaf-like structure.

Bars: 0.5 mm. S: sepals, Pe: petals, Pi: pistils.

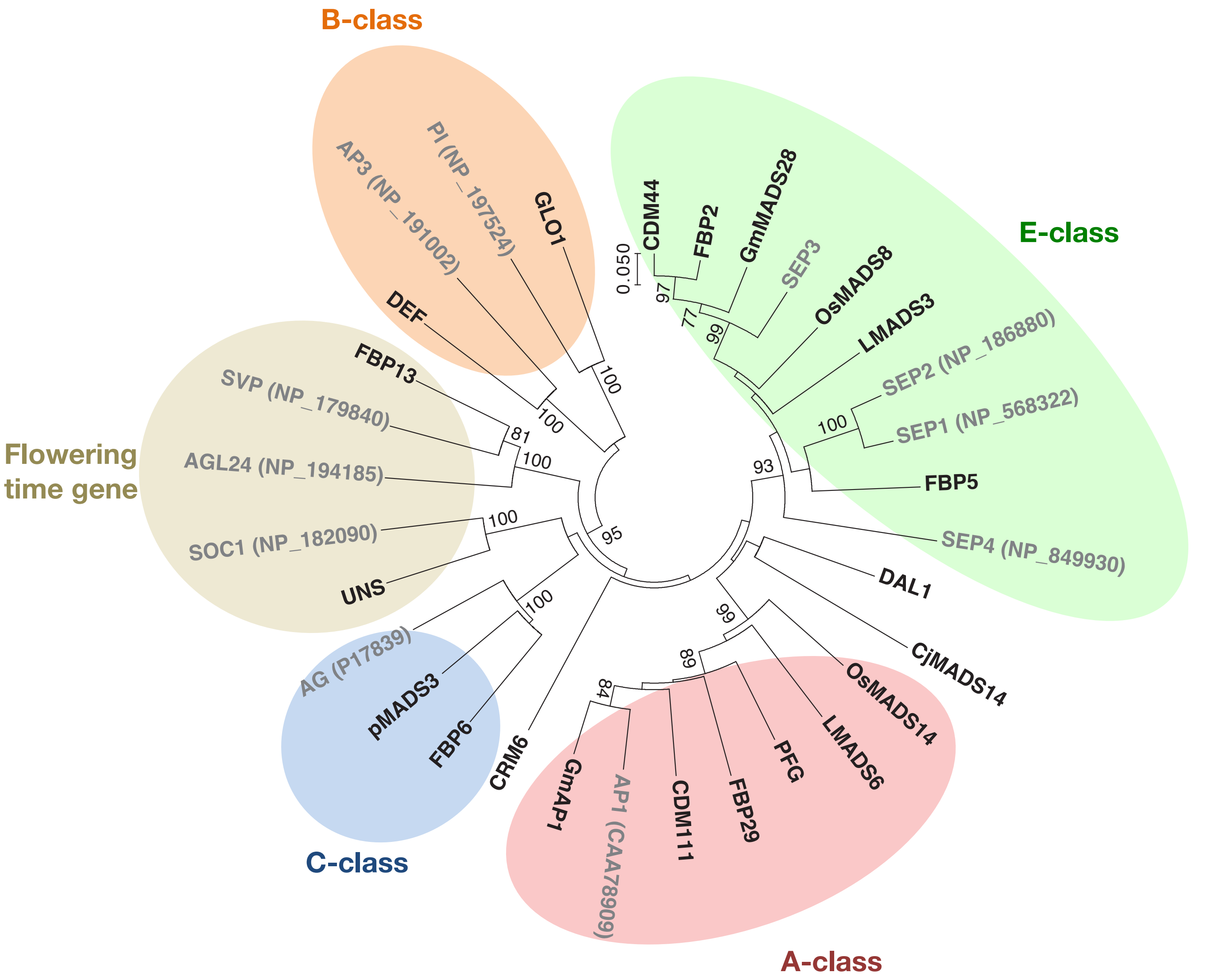

Supplementary Figure S5. Unrooted phylogenetic tree of the MADS domain transcription factors (MTFs) used in the present study. The tree was constructed based on amino acid sequences of the MTFs with gene groups of ABCE-class MTFs and flowering time genes in *Arabidopsis thaliana.* Phylogenetic analysis of the sequences was performed using the neighbor-joining method with MEGA version 7.0.18 software (Kumar *et al.* 2016). Numbers at the nodes represent bootstrap values >70%, obtained for 1,000 replicates. Scale bar indicates 5% sequence dissimilarity. Accession numbers are given in Table 1 or in parentheses. MTFs of *A. thaliana* are shown in gray.

Supplementary Table S1. Sequences of the primers used in this study.

Primer name	Sequence (5'→3')	Target gene	Purpose	Reference
1301ALSV-newRest-F	GGACTCTTTCCGGGTGGAGACGCCC		Vector construction	This study
1301ALSV-newRest-R	TTTGGATCCAGCGCTCCTAGGGTCGACTGTGAAATCAGGGCCCTGACCTTCT		Vector construction	This study
ALSV-NewCodon-R	TTTGTCGACAGTAAAGTCTGGTCCTTGTCCCTCCAACAAATTTGGGTCAGATC TATGCAAACTAG		Vector construction	This study
ALSV2600F	AGTGCTTCCACTCGTTTTAC		RT-PCR	This study
ALSV3000R	GTAGCATGACCACTCATGAT		RT-PCR	This study
FBP2_to_pGADT7_F	ACCAGATTACGCTCATATGGGAAGAGGTAGAGTTGAG	*FBP2*	Y2H (pGADT7)	This study
FBP2_pGADT7_R	TGCCCACCCGGGTGGAATCAAGGCAACCAGCCAGCC	*FBP2*	Y2H (pGADT7)	This study
PFG_to_pGADT7_F	ACCAGATTACGCTCATATGATGGGAAGAGGAAGAGTGCAG	*FBP29*	Y2H (pGADT7)	This study
PFG_pGADT7_R	TGCCCACCCGGGTGGAATTAGCCATTAAGATGGCGAAG	*FBP29*	Y2H (pGADT7)	This study
CDM44_to_pGADT7_F	ACCAGATTACGCTCATATGGGAAGAGGTCGAGTTGAGC	*CDM44*	Y2H (pGADT7)	This study
CDM44_pGADT7_R	TGCCCACCCGGGTGGAATCACTGATACCATCCTGG	*CDM44*	Y2H (pGADT7)	This study
CDM111_to_pGADT7_F	ACCAGATTACGCTCATATGGGAAGAGGTAAGGTACAG	*CDM111*	Y2H (pGADT7)	This study
CDM111_pGADT7_R	TGCCCACCCGGGTGGAATTAAGATGGAAAGCACCTC	*CDM111*	Y2H (pGADT7)	This study
OsMADS8_to_pGADT7_F	ACCAGATTACGCTCATATGGGGAGAGGGAGGGTGGAGC	*OsMADS8*	Y2H (pGADT7)	This study
OsMADS8_pGADT7_R	TGCCCACCCGGGTGGAATCAGGGTAGCCATGTCGGC	*OsMADS8*	Y2H (pGADT7)	This study
OsMADS14_to_pGADT7_F	ACCAGATTACGCTCATATGGGGCGGGGCAAGGTGCAGC	*OsMADS14*	Y2H (pGADT7)	This study
OsMADS14_pGADT7_R	TGCCCACCCGGGTGGAATTAGCCGTTGATGTGGCTC	*OsMADS14*	Y2H (pGADT7)	This study
LMADS3_to_pGADT7_F	ACCAGATTACGCTCATATGGAGCTGTCCGTGCTCTGTG	*LMADS3*	Y2H (pGADT7)	This study
LMADS3_pGADT7_R	TGCCCACCCGGGTGGAATTACATCCATGCTGGAGC	*LMADS3*	Y2H (pGADT7)	This study
LMADS6_to_pGADT7_F	ACCAGATTACGCTCATATGGGAAGAGGAAGAGTGCAG	*LMADS6*	Y2H (pGADT7)	This study
LMADS6_pGADT7_R	TGCCCACCCGGGTGGAATCATCCATTCACTCTAGAG	*LMADS6*	Y2H (pGADT7)	This study
CjMADS14_to_pGADT7_F	ACCAGATTACGCTCATATGGGGCGAGGGAAGGTTG	*CjMADS14*	Y2H (pGADT7)	This study
CjMADS14_to_pGADT7_R	TGCCCACCCGGGTGGAATCATAGCATATTCCAGC	*CjMADS14*	Y2H (pGADT7)	This study
DAL1_to_pGADT7_F	ACCAGATTACGCTCATATGGGGCGGGGTCGAGTCCA	*DAL1*	Y2H (pGADT7)	This study
DAL1_to_pGADT7_R	TGCCCACCCGGGTGGAATCAAACCCACCACCCTTGC	*DAL1*	Y2H (pGADT7)	This study
CRM6_to_pGADT7_F	ACCAGATTACGCTCATATGAGGAATGGCCTACTCAAGA	*CRM6*	Y2H (pGADT7)	This study
CRM6_to_pGADT7_R	TGCCCACCCGGGTGGAATCATTGTGCGAAGCCAAG	*CRM6*	Y2H (pGADT7)	This study
PNWB_to_pGBKT7_F	AGAGGAGGACCTGCATATGGATCCTAAGCTCCC	$PHYL1_{PnWB}$	Y2H (pGBKT7)	This study
pENTA_to_pGBKT7_R	CGACGGATCCCCGGGAATTTGTACAAGAAGCTGGGTCTAGAT	$PHYL1_{PnWB}$	Y2H (pGBKT7)	This study
PFG-F	CCTTCTTTCAAAGAAGGTGAAGGA	*PFG*	Real-time PCR	Himeno *et al.* (2011)
PFG-R	ATTCTGCTGCTCCCATTGAGTT	*PFG*	Real-time PCR	Himeno *et al.* (2011)
FBP29_rtF	GAGAGAGCGCTGCAAGAACA	*FBP29*	Real-time PCR	This study
FBP29_rtR	TCGTTTTGGCTGGAGATGG	*FBP29*	Real-time PCR	This study
PhAP2A-F	CGGCTCATCAAATTATCAGATTCA	*PhAP2A*	Real-time PCR	Himeno *et al.* (2011)
PhAP2A-R	AGGAGGTAAATTGGATTGCCATT	*PhAP2A*	Real-time PCR	Himeno *et al.* (2011)
PhGLO1-F	AATGAGGTTCTGAGGATGATGAGG	*GLO1*	Real-time PCR	Rijpkema *et al.* (2006*b*)
PhGLO1-R	CTTCGCCAATTTCTCCCATATTCC	*GLO1*	Real-time PCR	Rijpkema *et al.* (2006*b*)
PhDEF-F	AGAAGAAGGTCAGGAATGTGGAAG	*DEF*	Real-time PCR	Rijpkema *et al.* (2006*b*)
PhDEF-R	GTTGAAGGCGTAAGGCTAATATGC	*DEF*	Real-time PCR	Rijpkema *et al.* (2006*b*)
PMADS3-rt2F	AAACACGACAAATCGGCAAG	*PMADS3*	Real-time PCR	This study
PMADS3-rt2R	CTTCAGCATCACAGAGCACAGA	*PMADS3*	Real-time PCR	This study
FBP6-F	ACCATGCCGATTCCACAAGTA	*FBP6*	Real-time PCR	Himeno *et al.* (2011)
FBP6-R	CCTCTCCAACAATTTGCCTGTTA	*FBP6*	Real-time PCR	Himeno *et al.* (2011)
FBP2-F	GTGGCAGCAAAATGCACAAG	*FBP2*	Real-time PCR	Himeno *et al.* (2011)
FBP2-R	TTGGCCCTGCTCCTCCTAC	*FBP2*	Real-time PCR	Himeno *et al.* (2011)
FBP5-F	TATGATCCAGCTACTTCAAGCCAA	*FBP5*	Real-time PCR	Himeno *et al.* (2011)
FBP5-R	TTATGGTACATTCACAGCATCCAAC	*FBP5*	Real-time PCR	Himeno *et al.* (2011)
FBP13_rtF	ATGGTCCTACAGTTGAGGATGATTG	*FBP13*	Real-time PCR	This study
FBP13_rtR	GCTTCCCCATTTGAGTTTGCT	*FBP13*	Real-time PCR	This study
PhUNS_rtF	TCAGGGCGCGAAAGATG	*UNS*	Real-time PCR	This study
PhUNS_rtR	TTGATGCTTGTCTCTGTTGAAGG	*UNS*	Real-time PCR	This study
FBP25_rtF	GACAACTTAACAGCAGGGCAAG	*FBP25*	Real-time PCR	This study
FBP25_rtR	TATCAGCATCACAAAGAGTGGAGAG	*FBP25*	Real-time PCR	This study
PhGAPDH-F	ACCACTGTCCACTCACTTACTG	*GAPDH*	Real-time PCR	Rijpkema *et al.* (2006*b*)
PhGAPDH-R	TGCTGCTAGGAATGATGTTGAATG	*GAPDH*	Real-time PCR	Rijpkema *et al.* (2006*b*)
